# Casein Kinase 2-Interacting Protein-1 Alleviates High Glucose-Reduced Autophagy, Oxidative Stress, and Apoptosis in Retinal Pigment Epithelial Cells via Activating the p62/KEAP1/NRF2 Signaling Pathway

**DOI:** 10.1155/2021/6694050

**Published:** 2021-02-11

**Authors:** Xia Zhao, Jing Wang, Pei Li, Liying Tang, Yuzhi Bai

**Affiliations:** Department of Geriatrics, Beijing Chaoyang Hospital, Capital Medical University, Beijing 100043, China

## Abstract

**Background:**

Casein kinase 2-interacting protein-1 (CKIP-1) has been proved to be associated with complications of diabetes. Diabetic retinopathy is a main diabetic complication which usually leads to blindness. The current study aims to investigate the role of CKIP-1 in high glucose-treated retinal pigment epithelial (RPE) cells which is a component of blood-retinal barriers.

**Methods:**

The RPE cells, ARPE-19, are treated with high glucose to mimic the diabetic stimulation. CKIP-1 was overexpressed in ARPE-19 cells to evaluate its effects on autophagy, oxidative stress, and apoptosis induced by high glucose treatment, using Western blot, immunofluorescence, and flow cytometry assays, respectively.

**Results:**

CKIP-1 was expressed at a lower level in high glucose-treated cells than in normal glucose cells. Overexpression of CKIP-1 enhanced the Nrf2 translocation to the nucleus. Furthermore, high glucose-induced autophagy, oxidative stress, and apoptosis were inhibited after overexpression of CKIP-1. Also, CKIP-1 regulates the p62/Keap1/Nrf2 signaling, which might be the potential mechanism in this model.

**Conclusion:**

In conclusion, CKIP-1 may be a potential therapeutic target that protects RPE cells from injury and subsequent diabetic retinopathy induced by high glucose.

## 1. Introduction

Diabetic retinopathy (DR) is one of the common microvascular complications of diabetes leading to vision impairment and even blindness [1]. At the early stages of DR, ischemia, vascular leakage, and diabetic macular edema-induced central vision loss occur, accompanied by secondary angiogenesis and hemorrhage in the retina [2]. The blood-retinal barriers (BRB) mainly consisted of retinal endothelial cells and retinal pigment epithelial (RPE) cells, which can retain the completeness of retinal tissues. Alteration of BRB is essential for the development of retinal diseases [3]. Inflammation and oxidative stress are two risk factors for DR induction and progression [4, 5]. The RPE is a restrictive layer to prevent some molecules or charge transport and even maintain the balance of permeability of BRB [6]. Research has reported that continuing stimulation of hyperglycemia on RPE cells induces oxidative stress and cell apoptosis [7–9], which promotes BRB injury and subsequent DR progression.

Casein kinase 2-interacting protein-1 (CKIP-1), initially found as an interacting protein of casein kinase 2 (CK2) *α* subunit, is vital for apoptosis and oxidative stress [10]. A lot of diseases have been reported to be modulated by CKIP-1 expressions such as atherosclerosis, osteoporosis, and cardiac remodeling [11–13]. Only one study showed that CKIP-1 was associated with DR [14]. CKIP-1 upregulated Nrf2 by inhibiting Keap1 in hypoxia-induced cardiomyocyte injury [15]. Activating CKIP-1 promoted the Nrf2-ARE antioxidative pathway in the kidneys of high glucose-induced diabetic mice [16]. Upregulation of CKIP-1 also suppressed apoptosis and oxidative stress by inhibiting Keap1 and activating Nrf2/ARE signaling in hippocampal neurons [10]. The autophagic adaptor p62 was reported to physically interact with Keap1, an Nrf2 inhibitor, resulting in increased activation of Nrf2 and oxidative stress in hepatocellular carcinoma [17]. The p62-Keap1-Nrf2-ARE pathway plays a critical role in prion disease via regulating autophagy, oxidative stress, and mitochondrial dysfunction [18]. We supposed that CKIP-1 may also regulate oxidative stress, apoptosis, and autophagy by regulating p62/KEAP1/NRF2 signaling in high glucose-induced retinal pigment epithelial cells. This study identified a potential biomarker of BRB for the understanding of the correlation between epithelial activation and retinal diseases.

## 2. Materials and Methods

### 2.1. Cells and Treatment

Human RPE cell line, ARPE-19 (China Center for Type Culture Collection), was incubated in an incubator with 95% O_2_ and CO_2_ at 37°C and cultured in DMEM/F-12 (DF-12; Gibco, Thermo Fisher Scientific, USA) with 10% fetal bovine serum (FBS; Gibco, Thermo Fisher Scientific, USA). Cells were divided into three groups: low glucose normal control (5.5 mM, NG; DMEM, 11885092, Thermo Fisher Scientific, USA), high glucose (25 mM, HG; DMEM, 11965092, Thermo Fisher Scientific, USA), and mannitol negative control (5.5 mM glucose plus 20 mM mannitol, MG). To inhibit NRF2 expression, cells were exposed to 5 *μ*M ML385 for 24 h.

To study the role of CKIP-1 in HG-induced ARPE-19 cells, CKIP-1 was overexpressed in ARPE-19 cells by transfection with pcDNA3.1-CKIP-1 using Lipofectamine 3000 (Invitrogen) for 48 h. The ORF sequence of human CKIP-1 (GenBank No. NM_016274) cDNA was amplified by RT-PCR and subsequently cloned into vector pcDNA3.1 (Invitrogen, USA). The pcDNA3.1-empty vector plasmids were transfected as negative control.

### 2.2. RT-PCR

SuperScript™ IV One-Step RT-PCR System (Invitrogen, Thermo Fisher Scientific, and USA) was performed to evaluate the quantification of mRNA according to the manual and experimental protocol. The primers used for RT-PCR assay were as follows: CKIP-1, forward 5′-AATTCTGCGGGAAAGGGATTT-3′, reverse 5′-AACACCTCCTGACTGTTTTTCTC-3′ and *β*-actin, forward 5′-CTCCATCCTGGCCTCGCTGT-3′, and reverse 5′-GCTGCTACCTTCACCGTTCC-3′. The program thermal cycler at 60°C 10 min and 98°C 2 min for 1 cycle; 98°C 10s, 55°C 10 s, and 72°C 30 s for 40 cycles; and 72°C 5 min for 1 cycle. The relative quantification of mRNA levels was determined using the 2^−ΔΔCt^ method after being normalized to *β*-actin.

### 2.3. Western Blotting

Total proteins were obtained using cell lysis buffer (BioVision, USA). ExKine Nuclear and Cytoplasmic Protein Extraction Kit (KTP3001, Abbkine, USA) was used to separate and extract proteins from the nucleus and cytoplasm, respectively. The protein samples were separated by 10% SDS-PAGE gels and subsequently transferred to nitrocellulose membranes. The primary antibodies including CKIP-1 (ab91489, abcam, USA), LC3 (ab51520, abcam, USA), Beclin-1 (ab207612, abcam, USA), p62 (ab109012, abcam, USA), KEAP1 (ab196346, abcam, USA), NRF2 (ab76026, abcam, USA), cleaved-caspase3 (ab2303, abcam, USA), caspase3 (ab13847, abcam, USA), cleaved-caspase7 (ab256469, abcam, USA), and caspase7 (ab32522, abcam, USA) are incubated with membrane at 4°C overnight. HRP-conjugated goat anti-rabbit IgG secondary antibody (ab6721, abcam, USA) was incubated at room temperature for 2 h. GAPDH (ab181602, abcam, USA) was presented as an internal control of total protein. Lamin B2 (ab8983, abcam, USA) was used as the internal control of nuclear protein. Enhanced chemiluminescence reagent (Thermo Fisher Scientific, Inc., USA) was used to visualize the protein bands in a BioRad ChemiDoc XRS Imaging system (Hercules, USA).

### 2.4. Cell Viability Analysis

ARPE-19 cells were seeded in 96-well plates with 2000 cells/well in 100 *μ*l DF-12 medium with 10% FBS. After cells were adherent to the bottom of plates, ARPE-19 cells were starved with serum-free low glucose DMEM culture medium or high glucose DMEM medium or overexpressed by CKIP-1 and high glucose for 48 h. The cell viability was then evaluated by cell counting kit-8 assay (CCK-8; Beyotime, China). The optical density value was measured at 450 nm by a microplate reader (SpectraMax Gemini UVmax; Molecular Devices, USA).

### 2.5. Analysis of Oxidative Stress

1 × 10^6^ ARPE-19 cells per well in 6-well plates were cultured with 2 ml DF-12 medium with 10% FBS. Cells were treated as previously indicated. For ROS assay (Nanjing Jiancheng Bioengineering Institute, E004-1), cells after digestion were prepared into single-cell suspension and divided into negative, positive, and tested groups. The negative group was suspended with 0.01 mol/L PBS, and the positive group was suspended with DCFH-DA together with hydrogen peroxide to induce production of ROS. The tested groups were suspended with DCFH-DA alone. Cells in all groups were suspended into 1 × 10^6^∼2 × 10^7^ cells/ml. After suspension, cells were incubated in 37°C for 30 min. After that, cells were centrifuged under 1000 g for 5 min and washed with PBS once. After removal of the supernatant, cells were resuspended with PBS, and the fluorescence intensity was tested under the fluorescence spectrophotometer.

For SOD assay (Nanjing Jiancheng Bioengineering Institute, A001-3), cells after digestion were prepared into single-cell suspension and washed by PBS once before being smashed in the ultrasonic cell breaker. The mixture was centrifuged at 1000 g at 4°C for 5 min, and supernatant was used for the assay. Reagents and samples were mixed in a 96-well plate as described in the manual. The activity of SOD (U/ml) = 100% × ((OD_(control)_ − OD_(blank of control)_) − (OD_(test)_ − OD_(blank of test)_))/(OD_(control)_ − OD_(blank of control)_).

For MDA assay (Nanjing Jiancheng Bioengineering Institute, A003-1), cells after digestion were prepared into single-cell suspension and washed by PBS once before being smashed in the ultrasonic cell breaker. The mixture was centrifuged at 1000 g at 4°C for 5 min, and the supernatant was used for the assay. Reagents and samples were mixed in a 1.5 ml tube as described in the manual. After mixing, the mixture was heated at 95°C for 40 min and cooled down to room temperature. After centrifugation at 4000 g for 10 min, the supernatant was read at 532 nm. The OD values of the standard and test were corrected by OD of blank. Solutions 1, 2, and 3 were provided by the kit.

### 2.6. Cell Apoptosis

Cell apoptosis was determined by an Annexin V-FITC-PI apoptosis detection kit (Vazyme, China). Cells were harvested after treatment with indicated time at a concentration of 1–5 × 10^5^ cells. 100 *μ*l 1 × binding buffer was added to resuspend the cells. 5 *μ*l of V-FITC and 5 *μ*l of PI staining were gently added into the cells and incubated in the dark. After 15 min of incubation, 400 *μ*l 1 × binding buffer was added and mixed, and cells were then detected in 1 h. The Annexin V-FITC-positive and PI-negative cells were recorded as early apoptotic cells; the Annexin V-FITC-positive and PI-positive cells were recorded as later apoptotic cells. The apoptotic cells included the early apoptotic cells and later apoptotic cells.

### 2.7. Immunofluorescence

1 × 10^4^ ARPE-19 cells were plated on a coverslip and treated as previously indicated. 4% paraformaldehyde was used to fix the cells for 10 min, and 0.1% Triton X-100 was used to permeabilize the cells for 5 min. The goat serum was used to block for 1 h, and rabbit anti-LC3-II mAb (Alexa Fluor 488 conjugate; Cell signaling, #3868) was incubated with cells overnight at 4°C. 4′, 6′-diamidino-2-phenylindole (DAPI) was used to stain the nuclei for 3 min.

### 2.8. Statistical Analysis

The data are presented as mean ± SD of the number of determinations. Statistical difference analysis was calculated using GraphPad Prism 6.0 software. The differences in multiple groups were detected using one-way ANOVA with Tukey's test. *P* value less than 0.05 was treated as a significant difference.

## 3. Results

High glucose treatment triggered the reduction of CKIP-1 levels and the activation of oxidative stress and autophagy in retinal pigment epithelial cells.

**Figure 1:**
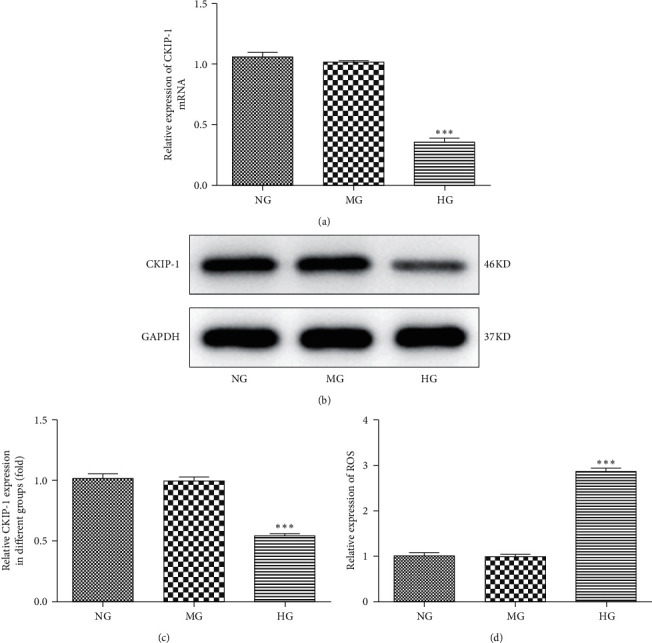
CKIP-1 expression is reduced in high glucose-triggered ARPE-19 cells. The mRNA (a) and protein (b) and (c) expressions of CKIP-1 were detected by RT-PCR and Western blots, respectively. (d) ROS generation was measured by the ROS kit. ^*∗∗∗*^*p* < 0.001 vs. NG. NG, normal glucose (5.5 mM); MG, mannitol (20 mM); HG, high glucose (25 mM).

**Figure 2:**
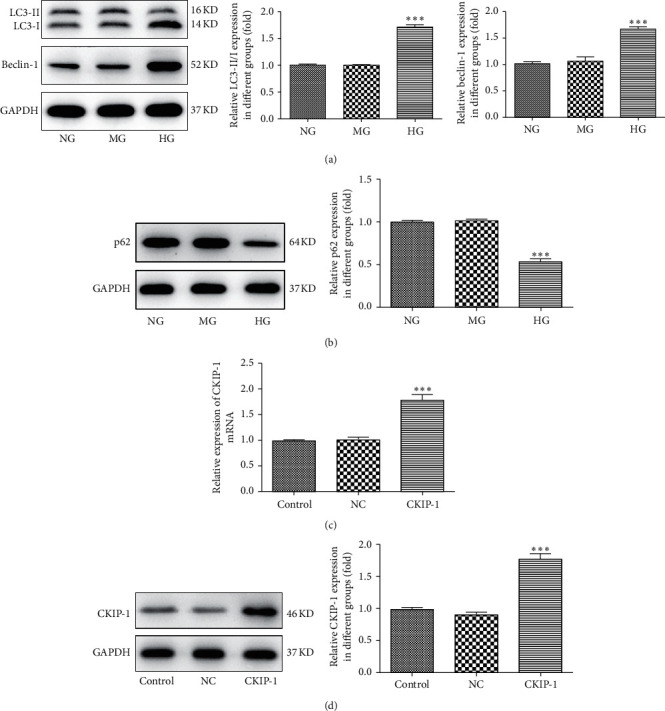
High glucose induced activation of autophagy in ARPE-19 cells. (a) Expression levels of the autophagic marker LC3 and Beclin-1 were determined by Western blots. (b) The expression of the autophagic substrate p62 was determined by Western blots. The transfected efficacy of CKIP-1 was evaluated by RT-PCR (c) or Western blots (d). ^*∗∗∗*^*p* < 0.001 vs. NG; ^*∗∗∗*^*p* < 0.001 vs. control. NG, normal glucose (5.5 mM); MG, mannitol (20 mM); HG, high glucose (25 mM); NC, pcDNA3.1 vector; CKIP-1, pcDNA3.1-CKIP-1.

To investigate the relationship between CKIP-1 and DR, we first detected the expression of CKIP-1 in high glucose-triggered ARPE-19 cells. Data showed that the mRNA expression level of CKIP-1 was downregulated after treatment of high glucose for 48 h (Figure 1(a), ^*∗∗∗*^*p* < 0.001). Similarly, the protein expression was also decreased in high glucose-treated ARPE-19 cells (Figures 1(b) and 1(c), ^*∗∗∗*^*p* < 0.001). Furthermore, high glucose-induced ROS expression, which indicated the activation of oxidative stress (Figure 1(d)). As LC3-II/I and Beclin-1 expression levels were increased and p62 expression was reduced, high glucose treatment on ARPE-19 cells dramatically promoted the autophagic flux (Figures 2(a) and 2(b), ^*∗∗∗*^*p* < 0.001). To further investigate the effects of CKIP-1 in DR, CKIP-1 was overexpressed in ARPE-19 cells (Figures 2(c) and 2(d), ^*∗∗∗*^*p* < 0.001).

CKIP-1 regulates the p62/KEAP1/NRF2 signaling in high glucose-triggered ARPE-19 cells.

**Figure 3:**
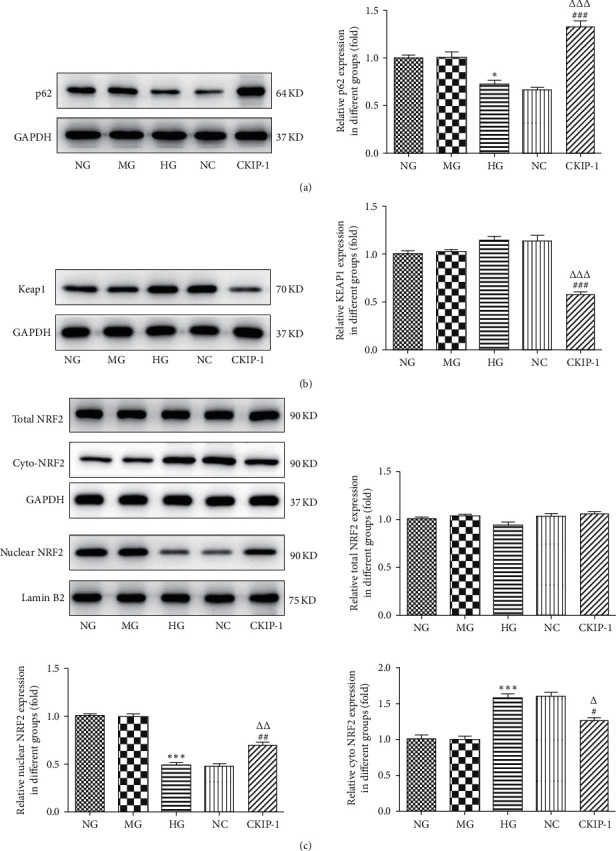
CKIP-1 activated the p62/Keap1/Nrf2 signaling pathway. (a) p62 expression was determined by Western blot in different groups. (b) Keap1 expression was determined by Western blot. (c) The expression of Nrf2 in whole cells or in the nucleus and cytosol. Lamin B2 and GAPDH were performed as the loading control of nuclear, whole cells, and cytoplasm, respectively. ^*∗*^*p* < 0.05 and ^*∗∗∗*^*p* < 0.001 vs. NG. ^#^*p* < 0.05, ^##^*p* < 0.01, and ^###^*p* < 0.001 vs. HG. ^Δ^*p* < 0.05, ^ΔΔ^*p* < 0.01, and ^ΔΔΔ^*p* < 0.001 vs. NC. NG, normal glucose (5.5 mM); MG, mannitol (20 mM); HG, high glucose (25 mM); NC, pcDNA3.1 vector negative control; CKIP-1, pcDNA3.1-CKIP-1.

Nrf2 translocates from the cytosol to the nucleus to respond to the oxidative stress, and p62-Keap1 interaction promotes the translocation of Nrf2 to the nucleus [19]. The results manifested that CKIP-1 overexpression enhanced the p62 accumulation in high glucose-treated ARPE-19 cells (Figure 3(a)). High glucose treatment did not affect the KEAP1 expression, but overexpression of CKIP-1 dramatically reduced the KEAP1 expression in ARPE-19 cells treated with high glucose (Figure 3(b)). In addition, the total Nrf2 levels were not changed in the cells both treated with high glucose or high glucose plus CKIP-1 overexpression. However, the level of nuclear Nrf2 was decreased in high glucose-treated cells, and CKIP-1 overexpression reenhanced the Nrf2 expression in the nucleus of ARPE-19 cells, but the cytoplasmic Nrf2 expression presented the opposite trend (Figure 3(c)), indicating that CKIP-1 might promote the translocation of Nrf2 to the nucleus.

CKIP-1 overexpression hinders the high glucose-induced autophagic flux in ARPE-19 cells.

**Figure 4:**
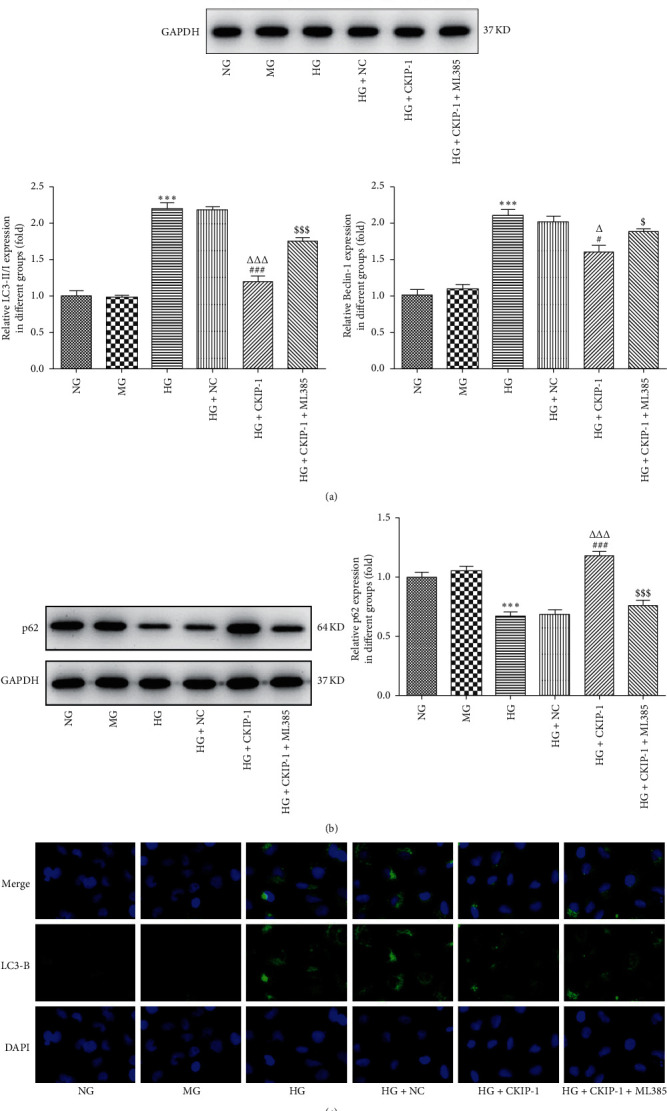
CKIP-1 inhibits high glucose-induced autophagy. (a) The LC3 and Beclin-1 expression was estimated by Western blots. (b) The protein expression of p62. (c) Immunofluorescence of LC3-II in ARPE-19 cells with different treatments. ^*∗∗∗*^*p* < 0.001 vs. NG. ^#^*p* < 0.05 and ^###^*p* < 0.001 vs. HG. ^Δ^*p* < 0.05 and ^ΔΔΔ^*p* < 0.001 vs. HG + NC. ^$^*p* < 0.05 and ^$$$^*p* < 0.001 vs. HG + CKIP-1. NG, normal glucose (5.5 mM); MG, mannitol (20 mM); HG, high glucose (25 mM); NC, pcDNA3.1 vector negative control; CKIP-1, pcDNA3.1-CKIP-1; ML385 (5 *μ*M), the Nrf2 inhibitor.

Due to high glucose induced autophagy, we next studied the effects of CKIP-1 on the autophagic pathway by Western blot. Overexpression of CKIP-1 prominently increased the p62 accumulation and inhibited the ratio of LC3-II/I (Figures 4(a) and 4(b)). In addition, the Beclin-1 expression was also decreased by CKIP-1 (Figure 4(a)). The results from immunofluorescence for the measurement of LC3-II were in accordance with the protein changes shown by Western blot (Figure 4(c)). Besides, the Nrf2 inhibitor, ML385, reversed the inhibitory effect of CKIP-1 on autophagy (Figure 4).

### 3.1. CKIP-1 Overexpression Suppresses High Glucose-Induced Oxidative Stress

**Figure 5:**
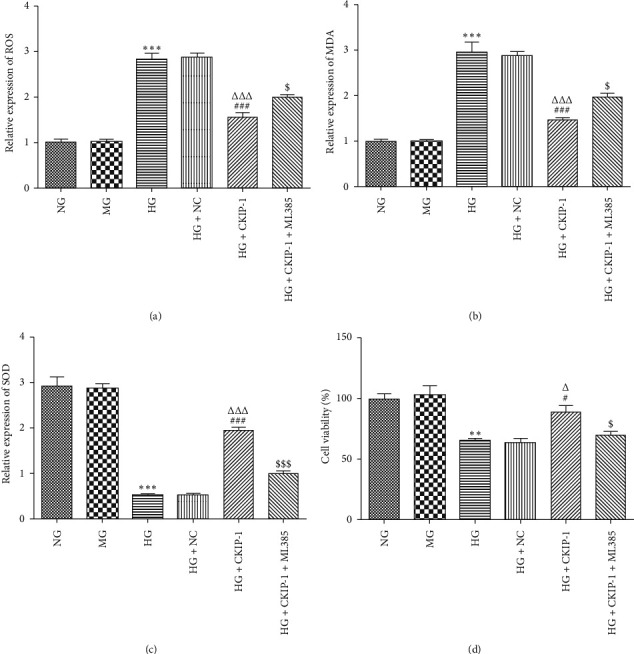
CKIP-1 suppresses oxidative stress and cell death induced by high glucose. (a) Relative production of ROS in different groups was evaluated by 2′,7′-dichlorofluorescein (DCFH) levels. (b) Relative expression levels of malondialdehyde (MDA) were evaluated by assay kit. (c) Relative activity of superoxide dismutase (SOD) was assessed by assay kit. (d) Cell viability was determined by the CCK-8 assay kit. ^*∗∗∗*^*p* < 0.001 vs. NG. ^###^*p* < 0.001 vs. HG. ^ΔΔΔ^*p* < 0.001 vs. HG + NC. ^$^*p* < 0.05 and ^$$$^*p* < 0.001 vs. HG + CKIP-1. NG, normal glucose (5.5 mM); MG, mannitol (20 mM); HG, high glucose (25 mM); NC, pcDNA3.1 vector negative control; CKIP-1, pcDNA3.1-CKIP-1; ML385 (5 *μ*M), the Nrf2 inhibitor.

Nrf2 cascade signaling can serve as an antioxidant response to regulate oxidative stress. To investigate the protective effects of CKIP-1, ROS was first accumulated by high glucose treatment. Then, the downregulated expression of ROS induced by CKIP-1 overexpression was observed in high glucose-induced ARPE-19 cells (Figure 5(a)). Increased CKIP-1 expression exerted a similar protective effect by dramatically decreasing the lipid peroxidation product, MDA levels, and recovered the antioxidant enzyme SOD level, revealing the role of CKIP-1 in alleviating tissue damage mediated by oxidative stress (Figures 5(b) and 5(c)). Furthermore, the Nrf2 inhibitor reactivated the oxidative stress in high glucose-treated cells transfected with pcDNA3.1-CKIP-1 (Figures 5(a) and 5(c)).

High glucose-triggered apoptosis is blocked in ARPE-19 cells with overexpression of CKIP.

**Figure 6:**
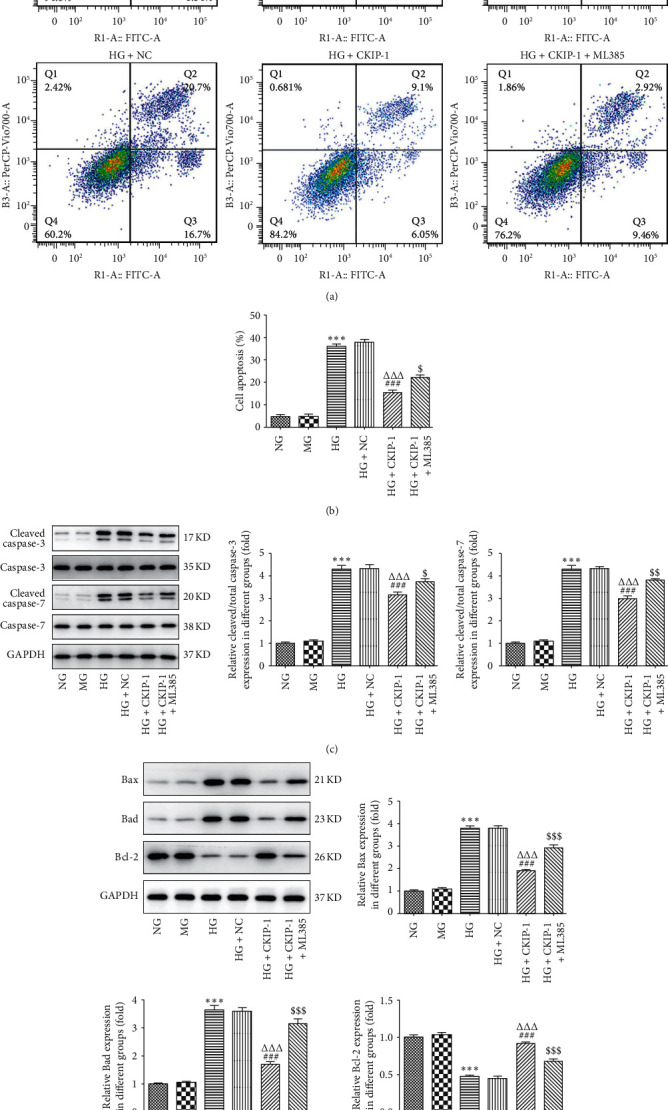
CKIP-1 alleviates apoptosis induced by high glucose. ((a) and (b)) The apoptotic cells were measured by flow cytometry and analyzed by flow Jo. ((c) and (d)) The apoptosis-associated markers were evaluated by Western blot. ^*∗∗∗*^*p* < 0.001 vs. NG. ^###^*p* < 0.001 vs. HG. ^ΔΔΔ^*p* < 0.001 vs. HG + NC. ^$^*p* < 0.05 and ^$$$^*p* < 0.001 vs. HG + CKIP-1. NG, normal glucose (5.5 mM); MG, mannitol (20 mM); HG, high glucose (25 mM); NC, pcDNA3.1 vector negative control; CKIP-1, pcDNA3.1-CKIP-1; ML385 (5 *μ*M), the Nrf2 inhibitor.

To further set forth the protective mechanism of CKIP-1 on RPE cells, we evaluated the relationship between cell death and CKIP-1. High glucose treatment for 48 h significantly induced cytotoxicity of ARPE-19 cells, which was reduced by CKIP-1 overexpression (Figure 5(d)). Furthermore, we found that CKIP-1 mainly reduced high glucose-induced apoptosis in ARPE-19 cells (Figures 6(a) and 6(b)). Concomitant with the apoptotic assay, Western blot showed similar results that high glucose enhanced the levels of proapoptotic proteins including cleaved-caspase3, cleaved-caspase7, Bax, and Bad inhibited by CKIP-1 (Figures 6(c) and 6(d)). Meanwhile, the level of antiapoptotic protein Bcl-2 was enhanced by CKIP-1 overexpression (Figure 6(d)). Therefore, the apoptosis induced by high glucose is blocked in ARPE-19 cells with overexpression of CKIP.

## 4. Discussion

Researchers have found that activation of CKIP-1 protects against diabetic renal fibrosis [20, 21]. Besides, evidence supported that CKIP-1 was dramatically downregulated in DR tissues and high glucose-treated human retinal endothelial cells [14]. The blood-retinal barrier (BRB) is composed of the tight junctions of the inner retinal endothelial cells and the outer retinal pigment epithelial cells, which regulate ion, protein, and water flux in and out of the retina [3]. BRB breakdown is thought to be one of the characteristics of DR, and the pathology is mainly associated with oxidative stress and inflammation [22]. In the current study, CKIP-1 was also downregulated in retinal pigment epithelial (RPE) cells under high glucose condition. Furthermore, overexpression of CKIP-1 in high glucose-treated RPE cells enhanced the ROS and MDA generation, as well as reduced SOD activity. Hence, we found that overexpression of CKIP-1 suppressed apoptosis. In DR progression, oxidative stress, endoplasmic reticulum stress-induced expression of death receptors, and mitochondrial damage are the major reasons that initiate apoptosis-related cell death [23]. The hyperglycemia promotes overproduction of ROS which induces mitochondrial dysfunction and apoptosis [24]. The results revealed the role of CKIP-1 in oxidative stress and apoptosis in DR progression, providing evidence that CKIP-1 can be a promising therapeutic target for DR therapy.

Autophagy can be a two-edged sword during DR progression. Activated autophagy can protect the cells from the occurrence of diabetes, endless autophagy; however, it will lead to autophagy dysfunction that elicits cell death [25, 26]. In the present study, high glucose induced the upregulation of ratio of LC3-II/I and the expression of Beclin-1 and downregulation of p62, indicating the activation of autophagy upon HG stimulation in RPE cells. However, CKIP-1 overexpression significantly decreased the expression of LC3-II/I and Beclin-1, but enhanced p62 expression. Emerging evidence has confirmed that autophagy is involved significantly in the progression of DR [27]. In the course of autophagy, LC3-I is transformed by the addition of a group of LC3-II which permits the combination of the protein to autophagosome membranes, and Beclin-1 plays a part to the initial autophagic vesicles development [27]. CKIP-1 has been reported to augment autophagy in steatotic hepatocytes [28]. Our research was the first to show that overexpression of CKIP-1 can inhibit the autophagy activation in high glucose-treated RPE cells. Excessive ROS production is found in mammalian cells to promote autophagy [29]. Besides, the antiapoptotic protein Bcl-2 inhibits the Beclin-1-dependent autophagy [30]. Overexpression of CKIP-1 suppressed not only the ROS level but also Bcl-2 expression, indicating that autophagy may mediate the role of CKIP-1 in high glucose-induced oxidative stress and apoptosis.

The p62 deficiency or overexpression in the autophagy process through transcriptional (in the nucleus) and posttranscriptional (in the cytoplasm) regulation has been investigated. Previous investigation has indicated that p62 assembly led to activation of Nrf2 through suppression of Keap1 [31, 32]. Our data showed that CKIP-1 overexpression enhanced the expression of p62 and nuclear Nrf2, but suppressed Keap1. In addition, the protective effects of CKIP-1 in high glucose-induced autophagy, oxidative stress, and apoptosis were reversed by the Nrf2 inhibitor, ML385, demonstrating that CKIP-1 shows that protective effects on high glucose-induced injury might be mediated by the p62/Keap1/Nrf2 signaling.

## 5. Conclusion

In conclusion, the current investigation proved that CKIP-1 overexpression could suppress high glucose-induced RPE cell autophagy, oxidative stress, and apoptosis by regulating the p62/Keap1/Nrf2 signaling pathway, which helps the exploration of new therapeutic strategy for DR.

## Data Availability

All data generated or analyzed during this study are included within the article.
